# Cell-free DNA integrity and complement C4d as novel liquid biopsy biomarkers for paraneoplastic and non-paraneoplastic autoimmune encephalitis

**DOI:** 10.3389/fimmu.2025.1640532

**Published:** 2025-09-17

**Authors:** Aigli G. Vakrakou, Maria-Evgenia Brinia, Anastasia Cheiraki, Anastasia Alexaki, Anna Papadopoulou, Giannis Vatsellas, Vasileia Kokala Dimitropoulou, Anastasia Derventzi, Vassilios C. Constantinides, Panos Stathopoulos, Foteini Boufidou, Leonidas Stefanis, Christine Stadelmann, Stefan Nessler, Elisabeth Kapaki, Constantinos Kilidireas

**Affiliations:** ^1^ Neuroimmunology Unit, 1st Department of Neurology, School of Medicine, Eginition Hospital, National and Kapodistrian University of Athens, Athens, Greece; ^2^ Greek Genome Center, Biomedical Research Foundation Academy of Athens, Athens, Greece; ^3^ 2nd Surgery Clinic, Aretaieio Hospital Athens Medical School, National and Kapodistrian University of Athens, Athens, Greece; ^4^ 1st Department of Propaedeutic Surgery, Hippokration General Hospital of Athens, Athens, Greece; ^5^ Neurochemistry and Biological Marker Unit, 1st Department of Neurology, School of Medicine, National and Kapodistrian University of Athens, Eginition Hospital, Athens, Greece; ^6^ Department of Neuropathology, University Medical Center Gottingen, Gottingen, Germany; ^7^ Department of Neurology, Henry Dunant Hospital Center, Athens, Greece

**Keywords:** cell-free DNA, C4d, encephalitis, tumor, IL-17A, IL-6, apoptosis, necrosis

## Abstract

**Background:**

Neuronal injury in autoimmune encephalitis (AE) may involve antibodies or T cells, with or without complement activation. Cell-free DNA (cf-DNA), released during cell death, and the complement split product C4d may reflect underlying tissue damage and immune activation. This study examines cf-DNA and C4d levels in the CSF and plasma of AE patients, focusing on differences between paraneoplastic and non-paraneoplastic subtypes.

**Methods:**

Thirty patients with AE (including paraneoplastic and non-paraneoplastic cases) and 18 healthy and disease controls were included. Total cf-DNA and cf-DNA integrity (cfDI), defined as the ALU-247/ALU-115 ratio, were measured in the CSF and plasma using quantitative polymerase chain reaction (qPCR). Interleukins IL-6 and IL-17A and the complement split product C4d were measured by ELISA. Clinical and radiological parameters were recorded.

**Results:**

CSF cf-DNA levels were significantly elevated in AE patients compared to controls (*p* < 0.01). Patients with paraneoplastic AE showed higher cfDI values (*p* < 0.05), indicating a predominance of necrotic cell death. CSF C4d levels were markedly increased in AE patients, particularly those with tumors (*p* < 0.001). CSF C4d showed the highest diagnostic accuracy for detecting underlying tumors at AE diagnosis (AUC = 0.818). Elevated CSF ALU-115 levels (*p* = 0.025) were significantly associated with MRI-confirmed encephalitic lesions, while increased cfDI correlated with electroencephalogram abnormalities indicative of epileptiform activity, underscoring their potential as biomarkers of disease severity.

**Conclusions:**

Elevated CSF levels of necrotic cf-DNA and the complement split product C4d reflect heightened CNS tissue injury and inflammatory activity in AE, particularly in paraneoplastic cases. These biomarkers may serve as useful tools for early diagnosis, disease monitoring, and subtype differentiation in AE.

## 1 Introduction

Immune-mediated encephalitis includes the classic paraneoplastic encephalitis syndromes, often associated with antibodies against intracellular neuronal proteins (onconeural antigens), and the encephalitis syndromes associated with antibodies against neuronal cell surface/synaptic proteins involved in neuronal signaling and synaptic plasticity, often referred to as “autoimmune encephalitis” (AE) (1). While paraneoplastic encephalitis syndromes are almost always linked to an underlying tumor, autoimmune encephalitis (AE) syndromes may present with or without cancer, reflecting a variable association that ranges from low to moderate depending on the antibody involved. Recent advances in diagnostic criteria emphasize a spectrum of cancer risk associated with different antibody and clinical phenotypes, categorizing them into high- and intermediate-risk groups, thereby refining the clinical approach to these heterogeneous disorders (2–5).

An important aspect in the pathogenesis of AE is the autoantibody-mediated functional effects in the cells expressing surface antigens (e.g., reduced expression of antigen, receptor cross-linking, internalization and degradation, interruption of cellular communication) and the presence in the affected central nervous system (CNS) tissue of B cells and plasma cells, as well as microglial activation (6). In some severe cases with available pathological data, variable complement activation has been documented (7–9). Nevertheless, it is unclear to what extent each antibody (and its subclasses) has a higher propensity for triggering complement activation *in vivo*. In paraneoplastic encephalitis, antibodies are considered non-pathogenic, with no reported complement-activating properties, and the immunopathology is indicative of T-cell infiltration and significant neuronal loss (10). CSF analysis has shown, in a limited number of studies, that GAD65 antibody-positive and neuronal surface AE displayed increased levels of activated complement proteins within the CSF compartment (9). Despite advances in our understanding of AE immunopathogenesis, reliable fluid biomarkers that reflect tissue-specific injury and immune activation remain scarce, especially in distinguishing paraneoplastic from non-paraneoplastic forms.

The above tissue abnormalities may cause cell-free genomic DNA fragments, known as circulating cell-free DNA (cf-DNA), to enter circulation and the CSF. Cf-DNA constitutes externalized, fragmented DNA of various lengths found in body fluids and is the product of either programmed cell death, necrosis, or cell activation (11). The sequence of the circulating cf-DNA provides many advantages as it includes biological information regarding the tissue of origin and its pathologies, such as chromosomal rearrangements, microsatellite alterations, point mutations, insertions and deletions, multi-nucleotide polymorphisms, loss of heterozygosity, copy number variations, and epigenetic changes (12–14). During apoptosis, DNA is cleaved at internucleosomal regions, producing characteristic fragments of approximately 167–180 base pairs (bp)—the length of DNA wrapped around nucleosomes—along with multiple numbers such as dinucleosomes (~360 bp) and trinucleosomes (~540 bp), generating the well-known “apoptotic ladder” pattern (15–17). In contrast, necrosis and other non-programmed cell death pathways result in the release of longer and more heterogeneous DNA fragments, which are preferentially amplified by ALU-247 primers (18–20). Cf-DNA integrity (cfDI) is measured as a ratio of longer DNA to shorter DNA for *Arthrobacter luteus* (ALU) repeats (247 and 115 bp). The ALU repeats are the most predominant short interspersed repetitive sequences in the human genome, expanded to a copy number of more than one million copies/genome (21). These longer fragments are not exclusively derived from necrosis, as apoptotic processes can also produce multinucleosomal cf-DNA fragments. Therefore, the cf-DNA integrity index (cfDI = ALU-247/ALU-115) is widely used as a probabilistic indicator of a relative increase in longer cf-DNA fragments, which likely reflects a greater contribution from necrotic or other non-apoptotic cell death pathways rather than apoptosis alone (22, 23). Beyond oncology, cf-DNA integrity has begun to be explored in neurodegenerative and autoimmune diseases, where increased cfDI values have been associated with complex neuronal injury involving necroptosis, pyroptosis, and inflammatory extracellular trap formation (24). Although data in these fields remain relatively scarce, this emerging evidence highlights cf-DNA integrity as a promising biomarker in the study of diverse pathological cell death mechanisms (25–27).

Furthermore, DNA outside the nucleus can act as a damage-associated molecular pattern (DAMP), clearly shown in DNAse-deficient mouse models (28). Cell-free nucleic acids isolated from sera derived from patients with systemic autoimmune diseases have been shown to prime the activation of both NLRP3 (NOD-like receptor family pyrin domain containing 3) and AIM2 (absent in melanoma 2)-inflammasome in healthy monocytes, suggesting the inflammagenic potential of extracellular DNA (29, 30). Previous research suggests that elevated interleukin-6 (IL-6) levels in the CSF from individuals with AE indicate the level of neuroinflammation, and anti-IL-6 drugs (tocilizumab) have been proposed as treatment options in refractory cases (31, 32). Moreover, in anti-NMDAR encephalitis, patients with increased disease severity and poor prognosis had a higher CSF Th17 cell percentage and IL-17A levels (33, 34).

The present study aimed to assess quantitatively the presence of genomic cf-DNA in the CSF and plasma in various forms of AE associated or not with the presence of an underlying tumor, where cfDI could inform us about the type of tissue destruction and cell death. We further aimed to correlate aspects of cf-DNA biology with clinical and radiological parameters, inflammatory cytokines (IL-17A, IL-6), and complement C4d levels. These cytokines were selected due to their established roles in neuroinflammatory cascades and potential to interact with cf-DNA-induced immune activation pathways. Complement C4d is a stable marker of classical and lectin pathway activation, persisting longer than other fragments like C3a and C5a, which are rapidly cleared (35–37). Its established role in autoimmune and cancer-related inflammation supports its selection as a relevant biomarker in this study. Identifying such biomarkers could facilitate earlier diagnosis, guide immunotherapy escalation, and distinguish tumor-associated from idiopathic forms of AE—challenges that persist in current clinical practice.

## 2 Methods

### 2.1 Study population

A total of 48 adults (30 patients and 18 controls) were included in the study. All the patients recruited were hospitalized in the 1st Department of Neurology of the National and Kapodistrian University of Athens during the years 2012–2024. Written informed consent was provided by all subjects and/or their next of kin (in case of confusion). The study had the approval of the Scientific Board and Ethics Committee of Aeginition Hospital (428/26.5.22) and was conducted according to the ethical guidelines of the 1964 Declaration of Helsinki.

Thirty patients fulfilled the criteria for AE as defined by Graus et al. (38). Patients who had no detectable known autoantibody, either in the serum or CSF, were excluded.

Patients were subdivided into those with detectable antibodies to an intracellular antigen and amphiphysin antibodies (*n* = 15) and those with detectable antibodies to an extracellular antigen (*n* = 15). Specifically, patients with encephalitis were treatment-naive, and their CSF was sampled during the acute onset of the syndrome. CSF was negative for viral DNA and other pathogens, including tuberculosis and cryptococcus.

The control group consisted of a cohort of 18 age- and sex-matched individuals. Fourteen out of 18 were healthy controls undergoing minor surgery (such as hernia repair or knee joint surgery under spinal anesthesia), and 4 out of 18 were patients suffering from headaches (2 with normal pressure hydrocephalus and the other with non-specific white matter lesions). Prior studies indicate that minor surgical procedures under spinal anaesthesia typically do not cause significant or sustained elevations in inflammatory cytokines such as IL-6 (39–42). For plasma biomarker analysis, 12 controls and 15 patients had available samples. The control group presented no cognitive complaints and no neurological, psychiatric, or other major diseases. They all had normal cognitive function as assessed by a semistructured interview based on the Mini-Mental State Examination (MMSE).

### 2.2 Diagnostic evaluation

Demographic and clinical data at the initial presentation were collected in all cases retrospectively. The presence of underlying malignancies was also reported. All patients underwent a complete physical and neurological examination, evaluating focal and peripheral symptoms/signs, autonomic dysfunction, psychiatric symptoms, cognitive impairment, and movement disorders. A battery of neuropsychological tests was performed in all patients, which included a) MMSE, b) the Frontal Assessment Battery (FAB), c) the 5-word immediate and delayed recall (5WT), and d) the 15-point spontaneous and copy CLOX drawing (CLOX1 and 2, respectively) tests. The modified Rankin scale (mRS) clinical score was applied for the estimation of the symptom severity at the time of admission to the hospital for the first attack, as used before in patients with AE (43–45).

Paraclinical investigations at the time of the initial hospitalization of the patients included electroencephalogram (EEG); electromyography (EMG); 3 Tesla magnetic resonance imaging (MRI) of the brain; abdomen, chest, and pelvis; and computed tomography (CT) to rule out concurrent tumor presence. Total body positron emission tomography and computed tomography (PET-CT) was performed, if necessary, especially in patients with high-risk paraneoplastic antibodies. At the time of diagnosis, cancer was detected in 11 out of 30 patients. More specifically, one patient with anti-Ma2 antibodies was diagnosed with esophageal cancer. Two patients with NMDAR encephalitis were found to have cystic teratoma. Among those with anti-Yo antibodies, two patients were diagnosed with cancer—one with endometrial cancer and the other with prostate cancer. The patient with anti-Zic4 antibodies was diagnosed with mediastinal cancer. Additionally, five patients were found to have lung cancer: three with multiple antibodies (anti-CV2 and anti-Hu; anti-CV2 and anti-Yo; and anti-GAD, anti-Hu, and anti-CV2), one with anti-CV2, and one patient with anti-Hu antibodies.

MRI was indicative of encephalitis when there were hyperintense signals on T2-weighted fluid-attenuated inversion recovery sequences highly restricted to one or both medial temporal lobes (limbic encephalitis), or in multifocal areas involving gray matter, white matter, or both, compatible with demyelination or inflammation (38).

Standard commercially available assays detecting a panel of antibodies in the serum and/or CSF were used; antibodies against intracellular antigens (Hu, Ri, Yo, amphiphysin, CV2/CRMP5, Ma2/Ta, Zic-4, Tr [DNER], Recoverin, GAD65, SOX1) were identified with the immunoblot technique, whereas antibodies against extracellular (NMDAR, AMPAR, LGI1, Caspr-2, GABAb1R, GlyR) and Kelch-11 antigens were assessed with fixed cell-based assays.

### 2.3 Isolation of cell-free DNA and quantitative assessment in paired CSF and plasma samples

CSF and plasma samples were collected following standard protocols. CSF was obtained via lumbar puncture into sterile tubes and immediately placed on ice to minimize nucleic acid degradation. Plasma samples were collected in EDTA tubes and processed promptly. Both CSF and plasma samples were centrifuged at 1,500*g* for 10 min at 4°C to remove cellular debris. Supernatants were carefully transferred to new tubes without disturbing the pellet. The samples were either processed immediately or stored at −80°C, with minimal freeze–thaw cycles to preserve cf-DNA integrity (46).

Cf-DNA was isolated from the plasma and CSF (720 μL) of the patients, using the NucleoSpin^®^ cf-DNA XS Kit (MACHEREY-NAGEL, Germany), which isolates free nucleic acids from a relatively low initial sample amount (240–720 μL of plasma or CSF) and yields a sufficient amount of cf-DNA at the end of the procedure for downstream applications. Selected plasma and CSF samples were processed via the NucleoSnap cf-DNA kit (MACHEREY-NAGEL, Germany), requiring 1 to 3 mL of initial sample volume. Cf-DNA fragments were analyzed in Agilent 4150/4200 TapeStation (2 μL of cf-DNA). With this method, the cf-DNA recovered was tested for quality and chain integrity. Electropherogram analysis was performed with a High Sensitivity D100 TapeStation Kit using approximately 2 μL of eluted DNA from CSF. Due to low quantities of cf-DNA isolated from less than 1 mL of CSF, the cf-DNA was not evident on TapeStation. The absence of a DNA peak within the cell-free range (150–200 bp) did not preclude downstream processing with RT-PCR, as the concentration may be lower than the limit of detection of the TapeStation assay. For that purpose, in selected patients, we isolated cf-DNA from a greater starting amount of CSF (1–3 mL), which led to the characteristic patterns (peak sizes indicative of mononucleosomal fragments) (**Supplementary Figure 1**).

Cf-DNA was quantified using the quantitative real-time PCR technique (RT-PCR). Quantitative PCR (qPCR) was performed to estimate the copy numbers of two fractions of ALU repetitive elements (ALU-115 and ALU-247), and DNA integrity (cfDI) was calculated as the ratio of ALU-247/ALU-115. The reaction mixture for each RT-PCR consisted of a template sequence (1 μL of standard genomic DNA and cf-DNA), 0.2 mM of the forward and reverse primers, and 1× of SYBR Green Master Mix, with a total volume of 20 μL.

Cf-DNA was quantified by qPCR by comparison against standards of genomic DNA (gDNA) with known concentrations using primers targeting the nuclear-encoded housekeeping gene (β-globin) (β-globin-F: GTGCACCTGACTCCTGAGGAG, β-globin-R: CCTTGATACCAACCTGCCCAG), ALU sequences (ALU-115-F: CCTGAGGTCAGGAGTTCGAG, ALU-115-R: CCCGAGTAGCTGGGATTACA; ALU-247-F: GTGGCTCACGCCTGTAATC, ALU-247-R: CAGGCTGGAGTGCAGTGG) (22). Using gDNA at known concentrations (male DNA, Applied Biosystems, Foster City, CA, USA), we prepared serially diluted genomic DNA samples at various concentrations (10 ng/mL, 1 ng/mL, 100 pg/mL, 10 pg/mL, 1 pg/mL) as qPCR standards. Samples were run in triplicate to acquire the mean concentration of samples relative to the standard curve. After the preliminary experiments, we concluded that the proper RT amplification included the following: pre-cycling heat activation of DNA polymerase at 95°C, 3 min followed by 40 cycles of denaturation at 95°C 30 s, annealing at 60°C and 64°C for 30 s (β-globin and ALU sequences, respectively) and extension at 72°C for 30 s, and a final extension at 72°C for 10 min (StepOne Plus).

### 2.4 Determination of inflammatory cytokine levels and C4d in both the CSF and plasma

The levels of inflammation-associated cytokines in both the CSF and plasma were quantified using Sandwich ELISA kits (IL-6 and IL-17A, both from e-Bioscience, San Diego, USA) according to the manufacturer’s instructions (undiluted samples). C4d was quantified in plasma samples (1:100 dilution) and undiluted CSF of the respective cohorts using the C4d MicroVue™ EIA (Quidel, San Diego, USA) according to the manufacturer’s instructions.

### 2.5 Statistical analysis

Data were expressed as mean ± SD or the median (range). Statistical analyses were performed using SPSS version 20.0 (IBM Corp., Armonk, NY, USA). Independent-sample non-parametric tests were performed to compare the levels of CSF cf-DNA or inflammatory cytokines/complement between patients and controls. Subsequently, to test the sensitivity and specificity of the tested biomarkers, receiver operating characteristic (ROC) curves were applied. Correlations among the quantitative parameters were evaluated using Pearson’s test; correlations between mRS scores and quantitative parameters were assessed with Spearman’s test. A *p*-value <0.05 was regarded as statistically significant.

## 3 Results

### 3.1 Demographic and clinical features of patients with encephalitis

The demographic data and clinical features of AE patients (*n* = 30) are shown in **Table 1**. The control group had a mean age of 60 ± 10 SD and a female/male ratio of 1. The mean age of the AE cohort was 59 years, with 52% female patients. However, the mean age at disease onset was significantly higher in patients with intracellular antibodies (68.87 ± 11.29 years) compared to those with neuronal surface antibodies (49.33 ± 19.78 years, *p* = 0.0038). There was no significant difference in sex distribution between the groups (both constituting 53.3% of female patients). Of the 30 AE patients, 15 (50%) had antibodies targeting intracellular antigens, including Ma2 (1/30, 3.3%), Yo (3/30, 10%), Zic-4 (1/30, 3.3%), amphiphysin (1/30, 3.3%), GAD65 (2/30, 6.6%), Hu (1/30, 3.3%), KLHL-11 (1/30, 3.3%), and multiple intracellular antibodies (predominantly anti-Hu; 5/30, 16.6%). The remaining 15 patients (50%) had antibodies targeting neuronal surface/extracellular antigens: NMDAR (6/30, 20%), Caspr2 (4/30, 13.3%), GlyR (3/30, 10%), GABAA R (1/30, 3.3%), and LGI1 (1/30, 3.3%).

**Table 1:** Clinical manifestations and characteristics of autoimmune and paraneoplastic encephalitis.

Clinical data	Subcategory	Intracellular antibodies (*n* = 15), *n* (%)	Neuronal surface antibodies (*n* = 15), *n* (%)	Tumor association, *n*	*p*-value
Demographics	Number of patients	15 (50%)	15 (50%)	–	–
	**Age at disease onset (years), mean (SD)**	68.87 (11.29)	49.33 (19.78)	–	**0.0038**
	Sex/female	8 (53.3%)	8 (53.3%)	–	N/S
Type of antibodies included	Ma2/Ta	1 (3.3)	–	1/1	–
	Yo	3 (10)	–	3/3	–
	Zic-4	1 (3.3)	–	1/1	–
	Amphiphysin	1 (3.3)	–	0/1	–
	GAD65	2 (6.6)	–	0/2	–
	Hu	1 (3.3)	–	1/1	–
	KLHL11	1 (3.3)	–	0/1	–
	Multiple intracellular antibodies:				
	- GAD, CRMP-5/CV2, Hu	1 (6.6)	–	–	–
	- CRMP-5/CV2, Hu	1 (6.6)	–	–	–
	- CRMP-5/CV2, Yo	1 (6.6)	–	–	–
	- GlyR, Amphiphysin, Titin	1 (6.6)	–	–	–
	- GlyR and Recoverin	1 (6.6)	–	–	–
	- CASPR-2, GAD65, SOX1	1 (6.6)	–	–	–
	NMDAR	–	6 (20)	2/6	–
	Caspr-2	–	4 (13.3)	0/4	–
	GlyR	–	3 (10)	0/3	–
	GABAb1R	–	1 (3.3)	0/1	–
	LGI1	–	1 (3.3)	0/1	–
Clinical presentation at onset	**Cognitive decline**	1 (6)	8 (53.3)	–	**0.014**
	Memory deficits	2 (13.3)	5 (33.3)	–	N/S
	Spatial disorientation	2 (13.3)	2 (13.3)	–	N/S
	Altered level of consciousness	1 (6)	2 (13.3)	–	N/S
	**Change in behavior**	1 (6)	7 (46.6)	–	**0.035**
	Psychiatric symptoms	2 (13.3)	3 (20)	–	N/S
	Dysarthria	4 (26.6)	0 (0)	–	N/S
	**Seizures**	0 (0)	7 (46.6)	–	**0.006**
	Peripheral nerve involvement	1 (6)	0 (0)	–	N/S
Selected clinical signs	Autonomic dysfunction	1 (6)	1 (6)	–	N/S
	Parkinsonism	2 (13.3)	1 (6)	–	N/S
	Dystonia	3 (20)	1 (6)	–	N/S
	**Cerebellar ataxia**	5 (33.3)	0 (0)	–	**0.041**
	Orofacial and limb dyskinesia	1 (6)	1 (6)	–	N/S
	Opsoclonus	1 (6)	0 (0)	–	N/S
	Acquired hyperekplexia	0 (0)	2 (13.3)	–	N/S
	mRS median (min–max)	4 (1–5)	4 (3–5)	–	N/S
Structural abnormalities	T2/FLAIR hyperintensities	11 (73)	10 (66)	–	N/S
	Gadolinium enhancement	1 (6.6)	3 (20)	–	N/S
	EEG changes	5 (33)	10 (66)	–	N/S

SD, standard deviation; *n*, number; GAD, glutamic acid decarboxylase; KLHL11, Kelch-like protein 11; NMDA, N-methyl-D-aspartate; Caspr-2, contactin-associated protein-like 2; Gly, glycine; GABAA, gamma aminobutyric acid; LGI-1, leucine-rich glioma inactivated 1; mRS, modified Rankin score; EEG, electroencephalography.Bold values indicate statistically significant differences (p < 0.05).

Common presenting symptoms included psychiatric disturbances (33.2%), memory deficits (46.6%), and seizures (46.6%). Significant differences between antibody groups were observed for several symptoms: cognitive decline was more frequent in patients with neuronal surface antibodies (53.3% vs. 6%, *p* = 0.014), as were behavioral changes (46.6% vs. 6%, *p* = 0.035) and seizures (46.6% vs. 0%, *p* = 0.006). T2/FLAIR hyperintensities related to encephalitis were noticed in 50% of patients, and EEG abnormalities in 66.6%. Other movement symptoms included autonomic disturbances, Parkinsonism, dystonia, cerebellar ataxia, orofacial and limb dyskinesia, opsoclonus, and acquired hyperekplexia. Among movement disorders, cerebellar ataxia was significantly more common in the intracellular antibody group (33.3% vs. 0%, *p* = 0.041). Other signs, such as autonomic dysfunction, Parkinsonism, dystonia, orofacial/limb dyskinesia, opsoclonus, and acquired hyperekplexia, did not differ significantly between the groups. However, there were no significant differences in the modified Rankin scale (mRS) scores between the two groups (median mRS = 4) (**Table 1**). Overall, these data highlight distinct clinical phenotypes linked to antibody type, with intracellular antibodies associated with older age and cerebellar ataxia, and neuronal surface antibodies associated with seizures, cognitive decline, and behavioral symptoms.

### 3.2 Cell-free DNA and cell-free DNA integrity in the CSF of patients with autoimmune encephalitis

**Figure 1:**
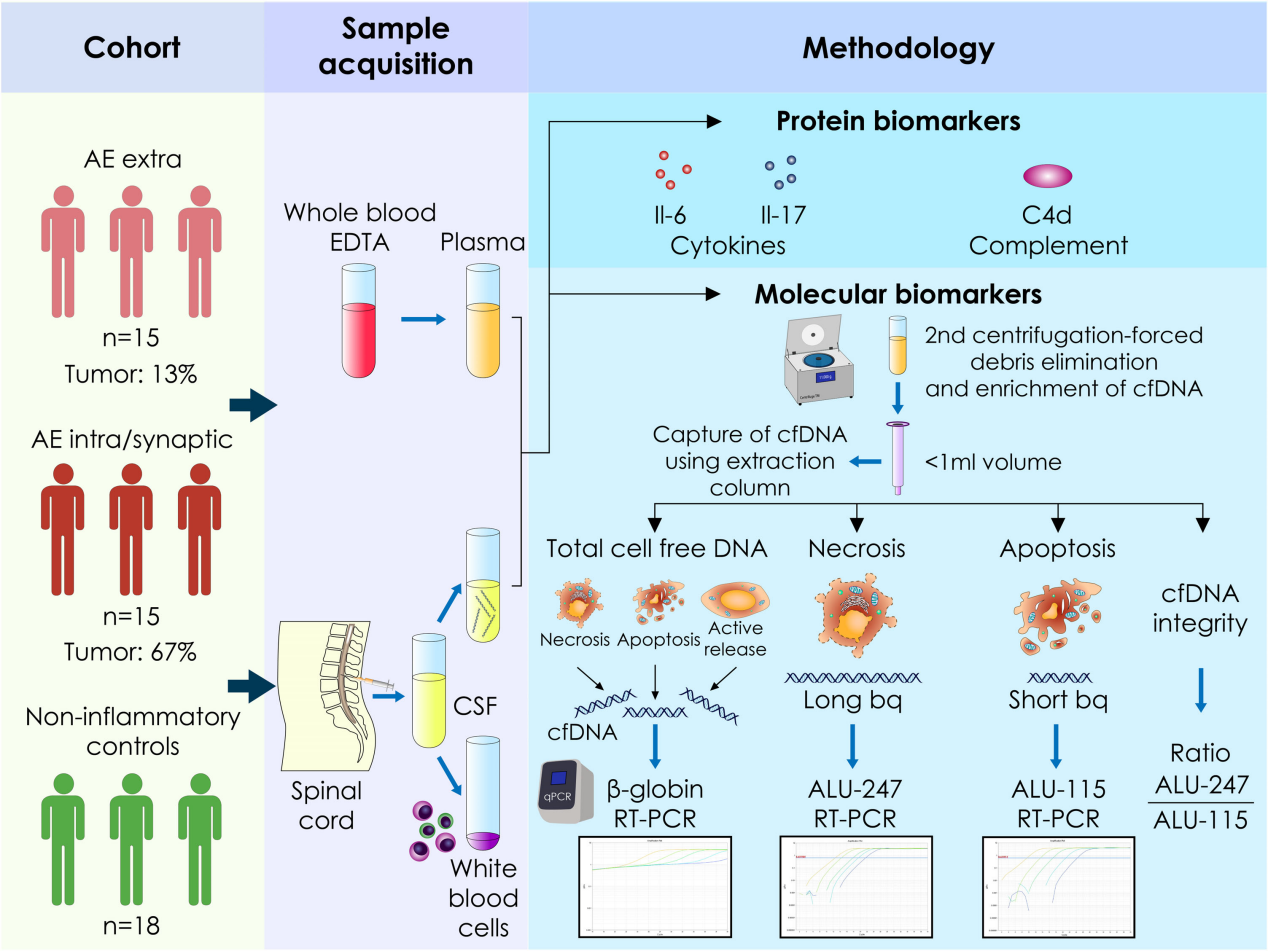
Schematic representation of the quantification of plasma and cerebrospinal fluid cell-free circulating DNA as well as the soluble cytokines IL-17A and IL-6 and the split C4d complement product in patients with autoimmune encephalitis and healthy controls. Raw data of the standard curves of the quantification of genes of interest as assessed by RT-PCR using human genomic DNA as template. Cf-DNA, cell-free DNA; bp, base pair; intra, intracellular antigens; extra, extracellular antigens; RT-PCR, reverse transcription-polymerase chain reaction.

**Figure 2:**
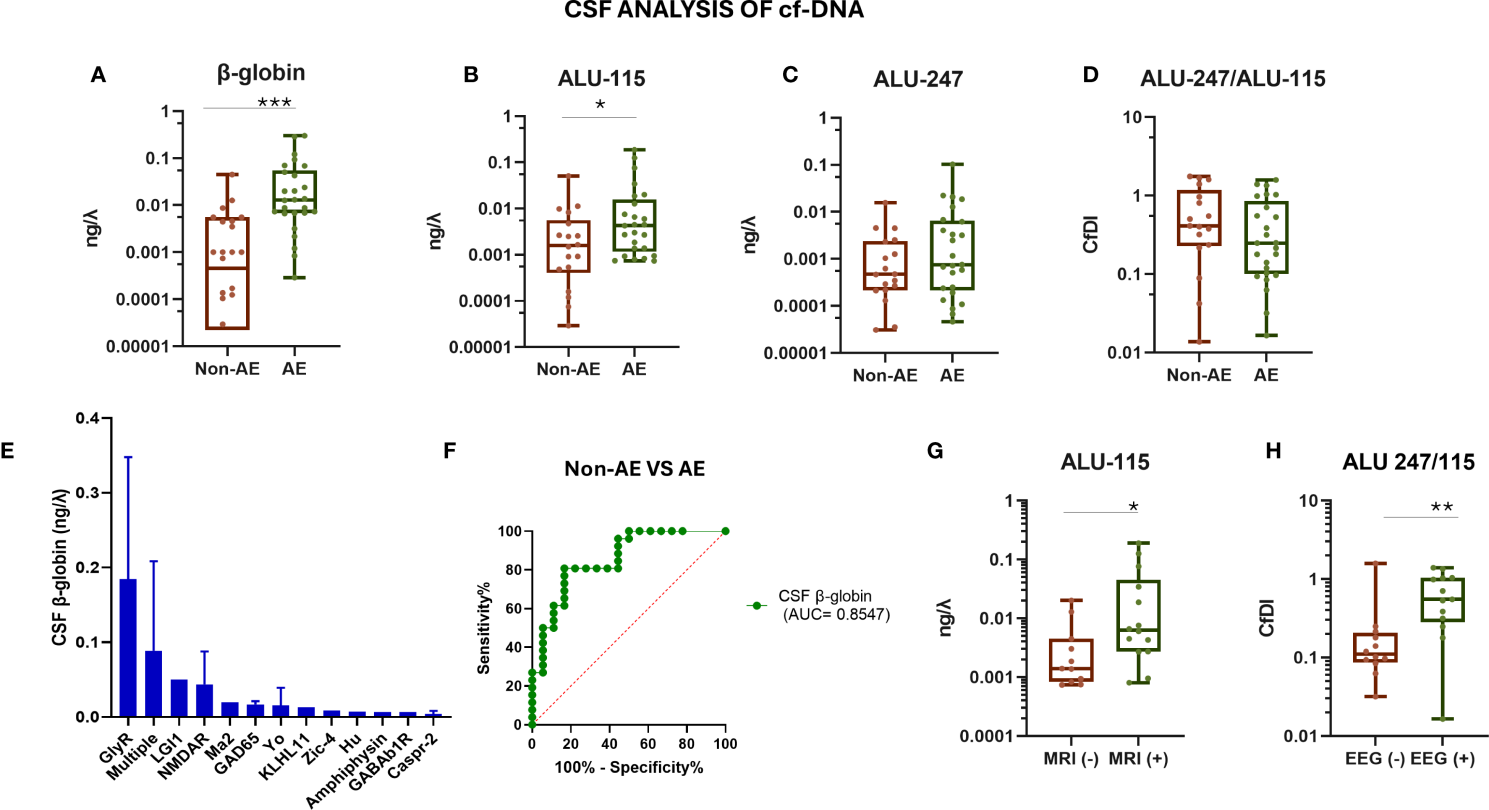
Evaluation of CSF cf-DNA in patients with autoimmune encephalitis and controls. **(A)** qPCR assessment of total CSF levels of cf-DNA by measuring the β-globin levels. **(B)** qPCR assessment of CSF levels of shorter (bp) size cf-DNA by measuring the ALU-115 levels. **(C)** qPCR assessment of CSF levels of longer (bp) size cf-DNA by measuring the ALU-247 levels. **(D)** Calculation of the ratio of ALU-247/ALU-115 (cfDI, cell-free DNA integrity). **(E)** cf-DNA (β-globin) among patients with different antibody-associated AE, measured as indicated above. **(F)** ROC curve assessing the sensitivity and specificity of measuring the β-globin levels in CSF as a biomarker for discrimination among patients and controls. **(G)** CSF ALU-115 levels in cf-DNA in two subgroups of AE patients stratified based on the presence or absence of MRI abnormalities indicative of the inflammatory process (MRI+). **(H)** CSF ALU-247/ALU-115 in the two subgroups of AE patients stratified based on the presence or absence of EEG abnormalities. Statistically significant results are depicted. *λ* = μL, *p < 0.05, ***p < 0.001. ROC, receiver operating characteristic curve; MRI, magnetic resonance imaging; AE, autoimmune encephalitis; non-AE, controls and disease controls; CSF, cerebrospinal fluid; EEG, electroencephalogram; cfDI, cell-free DNA integrity; cf-DNA, cell-free DNA. ** p < 0.01.

A schematic overview of the experimental workflow, including the quantification of plasma and CSF cf-DNA, cytokines (IL-17A and IL-6), and the C4d complement split product, as well as representative RT-PCR standard curves, is presented in **Figure 1**. AE patients showed significantly elevated CSF cf-DNA levels compared to controls (*p* < 0.0001) (**Figure 2A**). We next assessed the concentration of two repetitive elements in the CSF; thus, a short fragment (ALU-115) and a long fragment (ALU-247) were amplified and quantified. No significant differences in ALU-247 (necrosis-associated) levels between AE patients and controls were found, but a slight increase in ALU-115 (apoptosis-associated) levels (*p* < 0.0247) was noticed in patients with AE (**Figures 2B,C**). Consequently, cfDI, expressed as the ALU-247/ALU-115 ratio, exhibited wide variability but no group-level differences (**Figure 2D**). The highest levels of cf-DNA were detected in patients positive for GlyR, LGI1, and NMDAR antibodies and those displaying antibodies harboring antibodies against multiple intracellular antigens (**Figure 2E**). Levels of cf-DNA above 0.006 ng/μL could discriminate controls from AE patients with a sensitivity of 81% (95% CI: 62.12% to 91.5%) and a specificity of 83% (95% CI: 60.78% to 94.16%) (**Figure 2F**).

Patients with high CSF ALU-115 levels presented more frequently with MRI abnormalities indicative of encephalitis (as described in the *Methods*). Moreover, AE with increased ALU-247/ALU-115 ratio (cfDI) displayed abnormalities in the EEG (indicative of epileptic activity) (**Figures 2G, H**). Patients with the presence of CSF oligoclonal bands (OCBs) had higher levels of ALU-115 (*p* = 0.0141) (data not shown). Collectively, these findings suggest that specific elements of cf-DNA in CSF are associated with indices of disease activity.

### 3.3 Inflammatory cytokines and C4d levels in the CSF of patients with autoimmune encephalitis

**Figure 3:**
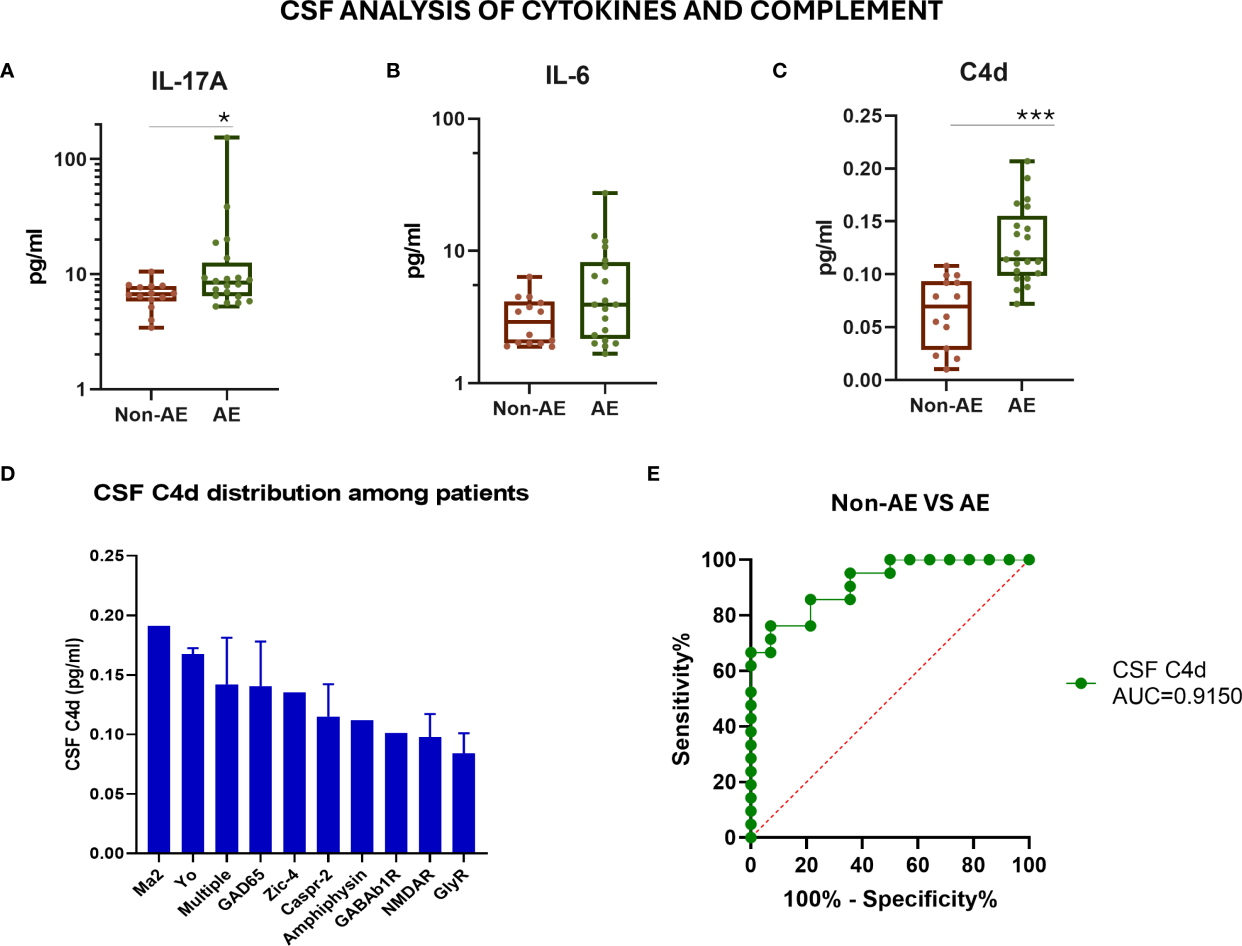
Evaluation of CSF inflammatory cytokine levels and complement C4d in patients with autoimmune encephalitis and controls. **(A)** Measurement of IL-17A cytokine levels in the CSF by ELISA. **(B)** Measurement of IL-6 cytokine levels in the CSF by ELISA. **(C)** Measurement of the complement component C4d levels in the CSF by ELISA. **(D)** CSF C4d distribution among AE patients with different antibody-associated AE. **(E)** ROC curve assessing the sensitivity and specificity of measuring the C4d levels in CSF as a biomarker for discrimination among patients and controls. Statistically significant results are depicted. *p < 0.05, ***p < 0.001. ROC, receiver operating characteristic curve; AE, autoimmune encephalitis; CSF, cerebrospinal fluid; AUC, area under the curve.

To further evaluate the role of humoral immunity and complement in AE pathogenesis, we measured the CSF levels of inflammation-related cytokines, IL-6 and IL-17A, and the C4d complement split product by ELISA. The CSF levels of IL-17A and the complement split product C4d were significantly elevated in AE patients compared to controls (both *p* < 0.001), whereas IL-6 levels showed no significant difference (**Figures 3A–C**). Notably, C4d was predominantly elevated in patients harboring multiple autoantibodies (**Figure 3D**). Among all tested biomarkers, CSF C4d exhibited the highest diagnostic performance in distinguishing AE patients from controls (AUC = 0.915) (**Figure 3E**). More specifically, a C4d concentration threshold above 0.09 pg/mL was indicative of AE with a sensitivity of 85.71% (95% CI: 65.6% to 95.02%) and a specificity of 78.57% (95% CI: 52.41% to 92.43%). **Table 2** summarizes the diagnostic performance of CSF biomarkers, including their AUC, sensitivity, specificity, and optimal cutoffs, providing a direct comparison of their accuracy in distinguishing AE patients from controls.

**Table 2:** Diagnostic performance of cerebrospinal fluid biomarkers in autoimmune encephalitis.

Biomarker	Method	AUC (95% CI)	Std. error	*p*-value	Optimal cutoff	Sensitivity (%)	Specificity (%)	Likelihood ratio
β-Globin	RT-PCR	0.855 (0.740–0.969)	0.058	<0.0001	0.0061	80.8	83.3	4.85
ALU-115	RT-PCR	0.684 (0.520–0.849)	0.084	0.041	0.0027	64.0	72.2	2.30
ALU-247	RT-PCR	0.602 (0.432–0.772)	0.087	0.257	0.0005	68.0	55.6	1.53
ALU-247/ALU-115	RT-PCR	0.617 (0.437–0.796)	0.091	0.205	0.317	60.0	70.6	2.04
IL-6	ELISA	0.657 (0.475–0.840)	0.093	0.124	3.848	55.0	71.4	1.93
IL-17A	ELISA	0.718 (0.545–0.891)	0.088	0.033	7.927	60.0	78.6	2.80
C4d	ELISA	0.915 (0.826–1.000)	0.045	<0.0001	0.09	85,71	78.57	4

AUC, area under the curve; CI, confidence interval; Std. error, standard error; RT-PCR, real-time polymerase chain reaction; ELISA, enzyme-linked immunosorbent assay; Sensitivity, true positive rate; Specificity, true negative rate; Likelihood ratio, ratio of true positive rate to false positive rate.

CSF C4d levels correlated significantly with standard markers of neuroinflammation, including CSF white cell count (*r* = 0.464, *p* = 0.0339), protein levels (*r* = 0.5242, *p* = 0.0147), and mRS disability scores (*r* = 0.507, *p* = 0.018) (**Supplementary Figure 2**). In contrast to the significant differences in the CSF compartment, a slight increase in plasma β-globin (*p* = 0.0138) and C4d (*p* = 0.1) was also observed in AE patients compared to controls, underscoring the localized nature of CNS complement activation (**Supplementary Figure 3**). No differences were found in the plasma with regard to the levels of inflammatory cytokines and cfDI, expecting a trend toward more C4d plasma levels in AE patients. As shown in **Supplementary Figure 4**, no strong or consistent correlations were observed between molecular and protein biomarkers across plasma and CSF compartments in AE patients. This highlights the compartmentalized nature of the immune response in autoimmune encephalitis and reinforces that CSF-specific molecular profiling offers more reliable insights into CNS-specific immune mechanisms in AE than plasma-based markers (**Supplementary Figure 4**).

### 3.4 CSF cell-free DNA integrity and C4d levels in paraneoplastic encephalitis

**Figure 4:**
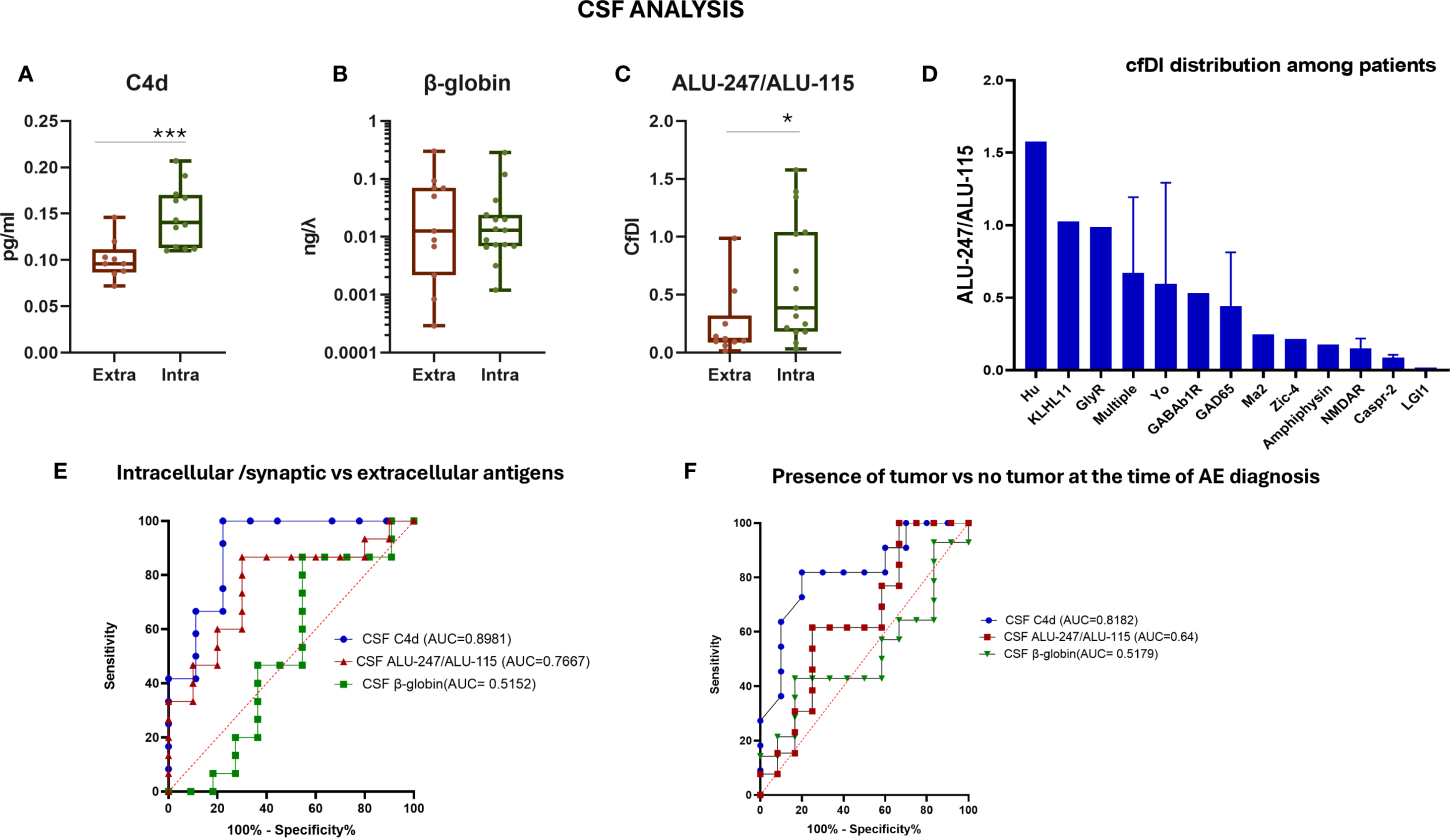
Evaluation of CSF cf-DNA in patients with autoimmune encephalitis separated into those with antibodies against intracellular antigens and extracellular antigens. **(A)** Measurement of C4d in the two patient subgroups by ELISA. **(B)** qPCR assessment of total CSF levels of cf-DNA by measuring the β-globin levels. **(C)** Calculation of the ratio of ALU-247/ALU-115 (cfDI). **(D)** CSF ALU-247/ALU-115 distribution among patients with different antibody-associated AE. **(E)** ROC curve assessing the sensitivity and specificity of measuring the ratio of ALU-247/ALU-115, C4d, and β-globin levels in CSF as a biomarker for discrimination among the two patient subgroups. **(F)** ROC curve assessing the sensitivity and specificity of measuring the ratio of ALU-247/ALU-115, C4d levels, and β-globin levels in CSF as a biomarker for discrimination among patients with or without an underlying tumor. Statistically significant results are depicted. *p < 0.05, ***p < 0.001. qPCR, quantitative real-time PCR; cfDI, cell-free DNA integrity; AE, autoimmune encephalitis; CSF, cerebrospinal fluid; intra, intracellular antigens; extra, extracellular antigens; ROC, receiver operating characteristic curve; AUC, area under the curve.

To inquire whether differences between AE patients and controls were driven by a particular subgroup, we divided AE patients into those with antibodies against intracellular antigens and those with antibodies against extracellular antigens. We found significantly increased CSF C4d levels and an increased ALU-247/ALU-115 ratio in AE patients with antibodies against intracellular antigens compared to those with antibodies against extracellular antigens (*p* < 0.001 and *p* < 0.05, respectively) (**Figures 4A–C**). The ALU-247/ALU-115 ratio was particularly increased in AE patients with antibodies against high-risk paraneoplastic antigens such as Kelch-11, Yo, and Hu (**Figure 4D**). CSF C4d levels displayed the best discriminating capacity between AE patients with antibodies against intracellular versus those with antibodies against extracellular antigens (**Figure 4E**).

Furthermore, we selected patients with a tumor diagnosis at the time of encephalitis diagnosis, including two patients with NMDAR encephalitis with teratoma, and compared them with those without concurrent tumors. ROC curve analysis of the various tested possible diagnostic biomarkers predictive of the presence of a tumor in patients with encephalitis at the time of disease diagnosis showed that C4d had the best discriminating capacity (AUC = 0.8182, 95% CI: 0.6310 to 1.000) (**Figure 4F**).

## 4 Discussion

Despite extensive research on immune-mediated encephalitis, significant gaps remain in our understanding of its pathogenesis, diagnostic biomarkers, prognostic indicators, and treatment response predictors (47, 48). At disease presentation, it is important to rule out the presence of an underlying tumor, as this knowledge could guide further therapeutic strategies. Moreover, in paraneoplastic encephalitis, the presence of an underlying tumor can reveal itself over the next years of follow-up (49). Therefore, the search for disease biomarkers is of great importance; however, due to the anatomical site of the abnormality and the presence of the BBB, the most suitable and commonly used methods involve non-invasive probing of the CNS (50, 51).

Our case–control study presents a novel understanding of the significance of CSF circulating tissue damage biomarkers such as cf-DNA and its components, as well as the C4d complement split product in immune-mediated encephalitis. Apart from total concentration, cf-DNA carries qualitative information, including tissue-specific methylation patterns and inflammatory potential. Increased cf-DNA concentration has been associated with cell death processes in degenerative and autoimmune disorders, ischemia, and trauma (52, 53). In amyotrophic lateral sclerosis (ALS), serum cf-DNA is elevated due to skeletal muscle-derived DNA, as revealed by methylation analysis, and more recently, mitochondrial DNA has been found elevated (54–56). In Alzheimer’s disease, plasma cf-DNA contains neuronal tissue-specific methylated genes such as LHX2 at CpG sites 1 and 5, suggesting potential as a peripheral neurodegeneration marker (57). In neuromyelitis optica spectrum disorder (NMOSD), serum cf-DNA is predominantly neutrophil-derived and induces type I interferon responses, illustrating inflammatory potential (58). These findings indicate that cf-DNA is a promising tool for biomarker development and provides mechanistic insights across CNS disorders, although dedicated CSF studies are required to determine its relevance.

cfDI, measuring the proportion of longer to shorter DNA fragments, can serve as a potential diagnostic and prognostic biomarker in various neoplastic diseases, autoimmune disorders, and myocardial infarction, but evidence for its application in autoimmune encephalitis remains limited (24, 59). Such analysis of cfDI in the present study revealed that patients with antibodies against intracellular antigens had more necrotic DNA released (increased ratio of ALU-247/ALU-115) in the CSF. Given the rarity and heterogeneity of autoimmune encephalitis, our study was necessarily based on a modest cohort size. While this context does not diminish the biological relevance of our observations, it underscores the need for cautious interpretation until findings are replicated in larger, independent series.

With regard to CSF C4d measurements, we found increased levels in patients with AE and antibodies against intracellular antigens with concurrent tumor presence. The histopathological hallmarks of paraneoplastic AE involve CD8 T-cell infiltration and cytotoxic T-cell-driven release of granzyme B, perforin, and Fas/Fas ligand, leading to variable levels of neuronal loss (10, 60). The terminal complex of complement C5b-9 has not been detected in the affected CNS tissue. Moreover, antibodies against intracellular antigens are generally not considered pathogenic. However, some *in vitro* evidence supports binding and internalization of some of these antibodies (anti-Yo and anti-Hu) by neurons, possibly contributing to subsequent neuronal injury (61–63). These results are controversial, and animal models of either active immunization with proteinic autoantigens or passive transfer of autoantibodies have not recapitulated the key features of the relevant paraneoplastic diseases. In biopsy or autopsy material, data regarding the precise type of neuronal cell death (apoptosis, necrosis, pyroptosis) and various complement components are lacking. Autopsy studies have shown that neuronal cell loss was prominent in five out of seven onconeural cases, two out of three GAD cases, and one out of seven surface antigen (VGKC-complex) cases. The most severe neuronal loss was found in one anti-Hu, one anti-Ma2, one anti-GAD, and one anti-VGKC complex case (6, 64). Evidence of IgG deposition close to neurons has been documented (65–69).

In AE with antibodies against surface antigens, autoantibodies confer functional perturbations, like blockage of receptors. Evidence of complement activation varies depending on the specific antigen (6). For example, complement can exacerbate antibody-mediated destruction in some patients with AE with anti-glycine receptor antibodies, anti-GABAb receptor encephalitis, and a small part of anti-Caspr-2 antibody-associated encephalitis (7, 70, 71). Also, the deposition of activated complement proteins has previously been observed in biopsy material from patients with LGI1 AE (8). Other autopsy and biopsy NMDAR encephalitis cases show deposits of complement in the tumor, but not in the brain, despite the local presence of IgG (70). The direct cytotoxicity of complement activation has been demonstrated in patients with GAD-Ab-associated encephalitis, who exhibited increased transcriptional levels of complement protein genes (C3, C4A, C4B) and elevated levels of activated complement proteins in the CSF (9).

In our study, elevated CSF levels of the complement split product C4d correlated with tumor presence in patients with encephalitis and with the presence of white blood cells in the CSF. The tissue origin of CSF C4d requires further investigation. No serum hypocomplementemia was present in any of the patients evaluated, suggesting no consumption of C4 in the periphery. In NMDAR patients, teratomas exhibited complement presence in their neural tissue, unlike the brain, which lacked complement deposition (72). C4d can be generated via the classical or the lectin pathway, and a multivalent ligand is required for activation. Cancer cells in clear cell renal cell carcinoma (ccRCC) tumors exhibited a complement-rich phenotype (73), while serum C4d has been correlated with tumor volume in malignant mesothelioma (74). So, it is hypothesized that tumor-derived C4d, possibly released to the plasma via microvesicles, is the main source of circulating levels. It is unknown if C4d could serve as a chemoattractant or if it evokes an increase in vascular permeability as C3a and C5a (75).

Several studies have shown that IL-6 is elevated in the CSF of patients with anti-NMDAR encephalitis but not in anti-LGI1 encephalitis, often accompanied by increased IL-17A and CXCL13, suggesting a subtype-specific pattern of Th17-driven inflammation (32, 34, 76, 77). IL-6, together with TGF-β, promotes Th17 differentiation via STAT3 signaling, and Th17 immunity has been implicated in AE pathogenesis (78). In our cohort, IL-6 levels were not significantly elevated, a finding that may reflect the predominance of non-NMDAR cases, the transient nature of IL-6 elevation, and the limitations of single time point sampling. These considerations align with previous reports that IL-6 changes in AE are both subtype-dependent and temporally variable, underscoring the need for larger, longitudinal, and subtype-stratified studies to define cytokine profiles more precisely.

We observed a lack of significant correlations between CSF and plasma biomarker levels in our cohort. This likely reflects compartmentalized immune responses within the CNS, which are not fully captured by peripheral blood measurements. This finding aligns with a recent systematic review and meta-analysis by Gigase et al. (2023), reporting a low pooled correlation (*r* ≈ 0.21) between blood and CSF inflammatory markers across neuroinflammatory diseases, with only modest correlations for cytokines such as IL-6 and TNF-α (79). These results emphasize that plasma biomarkers alone may be insufficient to comprehensively monitor CNS inflammation, highlighting the importance of CSF analysis for accurate assessment of disease activity in autoimmune encephalitis.

Our study, due to its retrospective nature, has several limitations. First, the relatively small cohort size, which becomes even more pronounced when patients are divided into subgroups, limits the statistical power and the generalizability of our findings. This is a common challenge in autoimmune encephalitis biomarker research, given the rarity and heterogeneity of the disease (80). Only antibody-positive AE patients were included; thus, applicability to seronegative cases remains to be evaluated. Healthy and non-inflammatory controls were limited due to sample availability, and future studies should expand control groups, potentially including patients with infectious encephalitis, to further validate specificity. Second, we assessed CSF biomarkers at a single time point, precluding evaluation of longitudinal cf-DNA changes during the disease course. Pathology studies addressing the types of cell death in the CNS in paraneoplastic encephalitis are still lacking. Moreover, our patients with paraneoplastic disease have an underlying tumor at disease diagnosis, and it is currently unknown if the aberrant necrotic DNA released could be part of the neoplastic tissue itself. This concern is partially mitigated by the localization of cf-DNA changes within the CSF compartment, suggesting a central rather than peripheral origin. Future pathology studies in AE patients are needed to explore the cell distribution of C4d in leptomeninges, subpial surfaces, and within and around vessels of the cortical and hippocampal gray matter.

We acknowledge that the presence of cancer is a significant confounder when interpreting cf-DNA levels in AE, as tumor-derived circulating tumor DNA (ctDNA) can substantially elevate total cf-DNA in the plasma and CSF (81, 82). In our cohort, 11 patients had paraneoplastic AE, consistent with the known association between certain tumors and autoimmune encephalitis (5). Although tumor-derived cf-DNA may contribute to the elevated cf-DNA observed in these cases, the marked cf-DNA increases detected in non-paraneoplastic patients support the notion that CNS injury and neuroinflammatory processes independently drive cf-DNA release. Disentangling tumor- versus CNS-derived cf-DNA is challenging but crucial, and future investigations incorporating tumor genotyping and analysis of tumor-specific mutations in cf-DNA are warranted to clarify the relative contributions of neoplastic and neuroinflammatory sources (83).

Collectively, our aim was to provide a proof-of-concept study demonstrating that cf-DNA integrity and C4d are elevated in paraneoplastic AE, where biomarkers are currently lacking and tissue injury and clinical severity are more prominent. Importantly, toward this, our workflow from CSF collection to cf-DNA extraction and quantification can be performed within ~5 h using minimal CSF (~720 µL). C4d measurement via ELISA requires only 1.5 h. In clinical practice, CSF total nuclear cf-DNA could be measured first in suspected AE (38). If elevated, additional biomarkers (cfDI and C4d) may indicate the presence of an underlying tumor or paraneoplastic process, enabling a rational biomarker-driven workflow for early decision-making. Additionally, our results could also be explored and open new avenues in seronegative AE, where the presence of cancer is not directly suggested by a known antibody.

## 5 Conclusions

In this study, we demonstrate that CSF cf-DNA levels are significantly elevated in AE patients, with cf-DNA integrity particularly increased in those with paraneoplastic etiologies. Our findings suggest that cf-DNA and its fragmentation patterns reflect underlying neuronal injury mechanisms and may serve as accessible biomarkers of tissue damage in AE. Moreover, the complement split product C4d emerges as a highly sensitive and specific biomarker for disease activity and tumor association, particularly distinguishing paraneoplastic from non-paraneoplastic cases.

These results highlight the potential utility of CSF cf-DNA and complement activation products as minimally invasive tools for improved diagnosis, prognosis, and monitoring in AE. Future longitudinal studies and mechanistic investigations are warranted to validate these biomarkers and to elucidate their role in AE pathophysiology, which could ultimately guide personalized therapeutic approaches.

## Data Availability

The original contributions presented in the study are included in the article/**Supplementary Material**, further inquiries can be directed to the corresponding author/s.

## Supplementary material

Supplementary Figure 1 Analytical specification of cell-free DNA in CSF. Two characteristic paradigms of DNA extracted from CSF (NucleoSnap column) of autoimmune encephalitis patients and analyzed using the Agilent 4200 TapeStation System. Two electropherogram profiles of cf-DNA CSF samples that represent differing quality dependent on the amount of low molecular weight DNA detected (white arrows, left: approximately 200bp and right: 170bp). Black arrows indicate peaks characteristic of high molecular weight genomic DNA.

Supplementary Figure 2 Correlation analysis of CSF C4d levels with various clinical and CSF parameters. mRS; modifying Rankin scale, WBC; white blood cells.

Supplementary Figure 3 Plasma levels of cell-free DNA, DNA integrity, inflammatory cytokines, and C4d between controls and patients with autoimmune encephalitis. Statistically significant results are depicted, *p<0.05. AE, autoimmune encephalitis; non-AE, controls and disease controls.

Supplementary Figure 4 Correlation matrix of all parameters in CSF and plasma measured in patients in all types of autoimmune encephalitis. In the correlation matrix the Spearman R is depicted, and color intensity corresponds to the magnitude of the correlation (negative: blue colors, positive: purple). C, cerebrospinal fluid; p, plasma; CFDI, cell free DNA integrity. Image was generated using Flourish (https://flourish.studio).
