# Supplementary figures and images for: Cell-free DNA integrity and complement C4d as novel liquid biopsy biomarkers for paraneoplastic and non-paraneoplastic autoimmune encephalitis
Source: Front Immunol. 2025 Sep 17;16:1640532. doi: 10.3389/fimmu.2025.1640532 (PMC12484147; doi:10.3389/fimmu.2025.1640532)

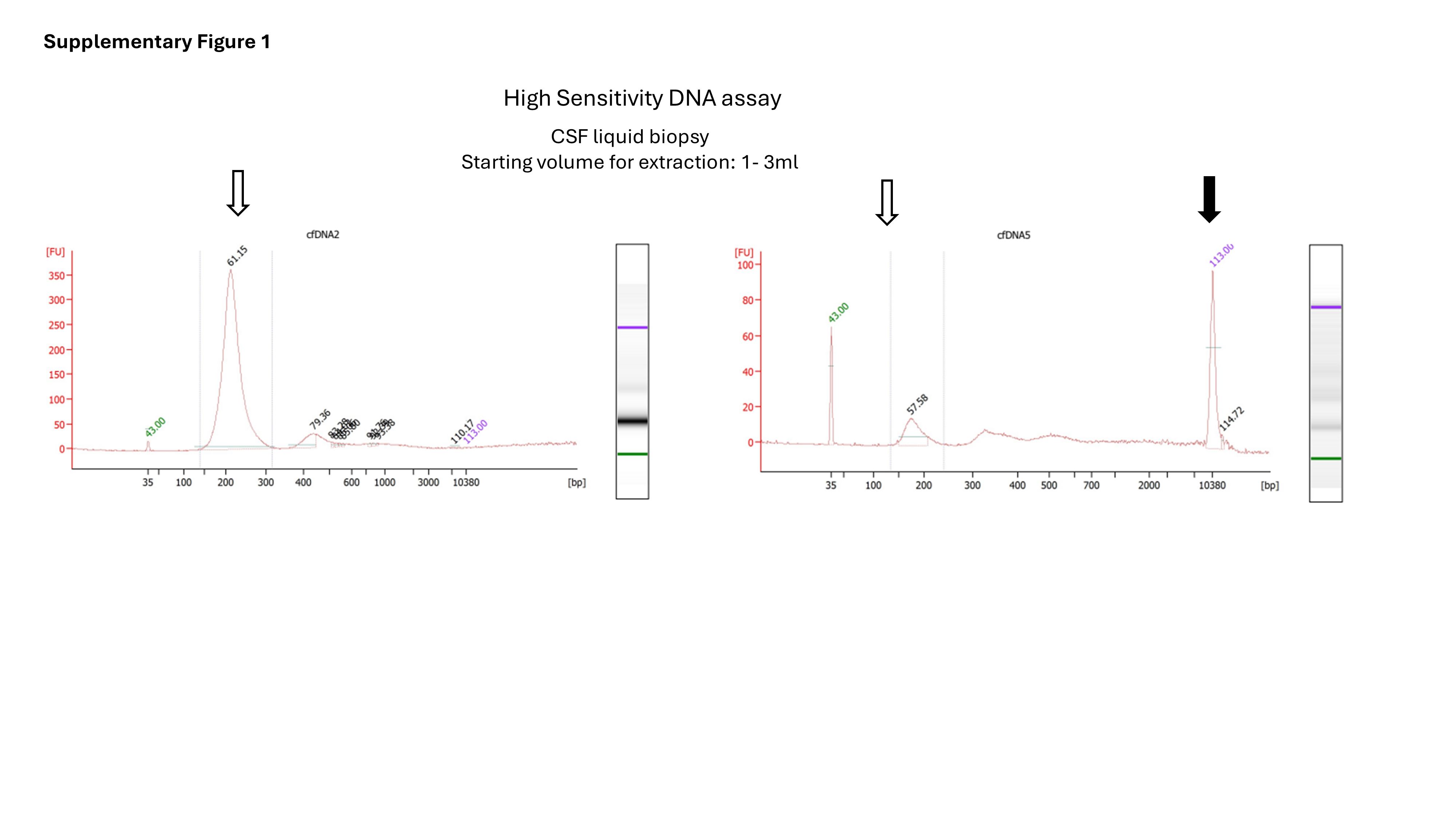

Supplement: Supplementary Figure 1 — Analytical specification of cell-free DNA in CSF. Two characteristic paradigms of DNA extracted from CSF (NucleoSnap column) of autoimmune encephalitis patients and analyzed using the Agilent 4200 TapeStation System. Two electropherogram profiles of cf-DNA CSF samples that represent differing quality dependent on the amount of low molecular weight DNA detected (white arrows, left: approximately 200bp and right: 170bp). Black arrows indicate peaks characteristic of high molecular weight genomic DNA. [file Image1.tif]

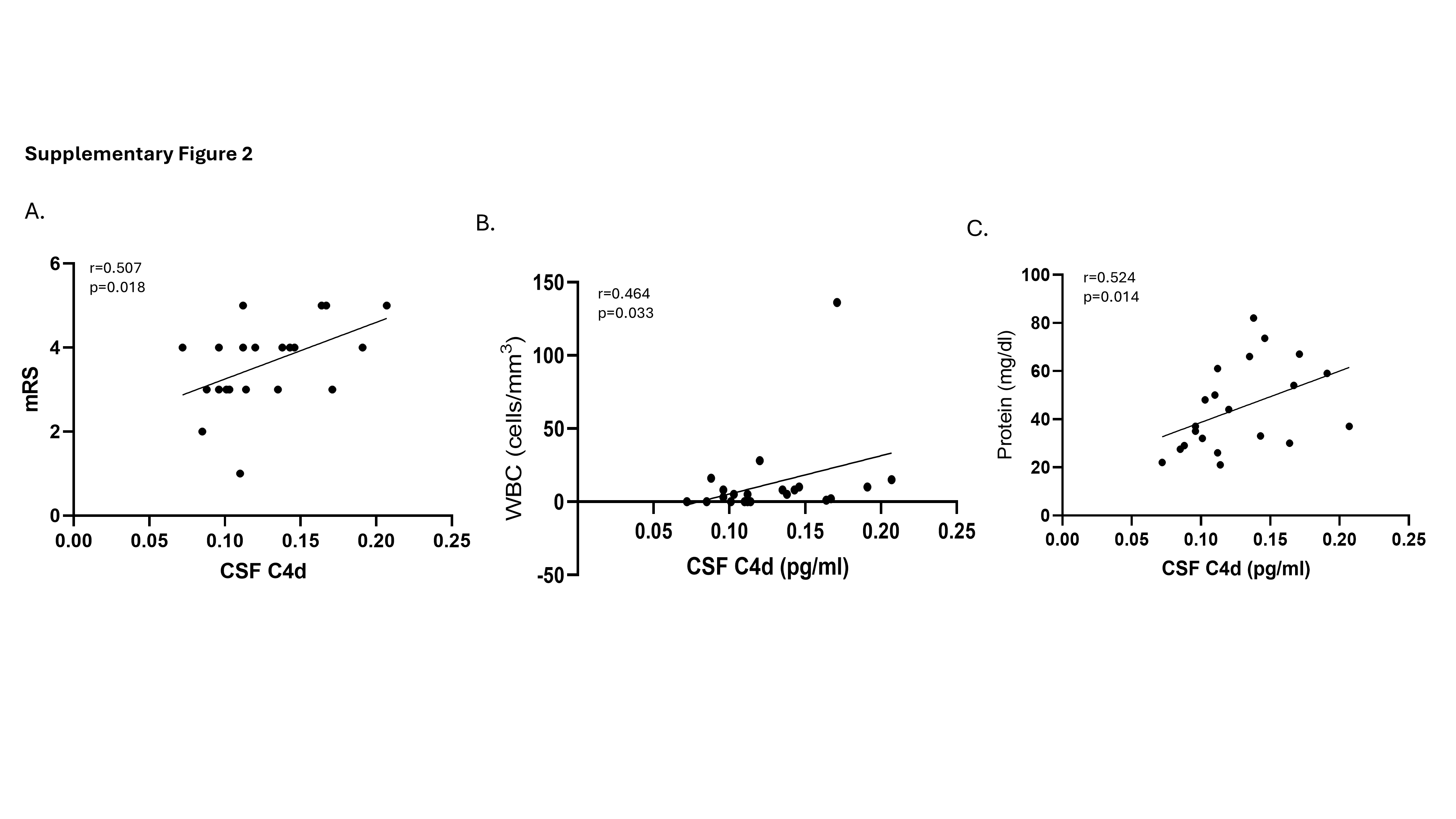

Supplement: Supplementary Figure 2 — Correlation analysis of CSF C4d levels with various clinical and CSF parameters. mRS; modifying Rankin scale, WBC; white blood cells. [file Image2.tif]

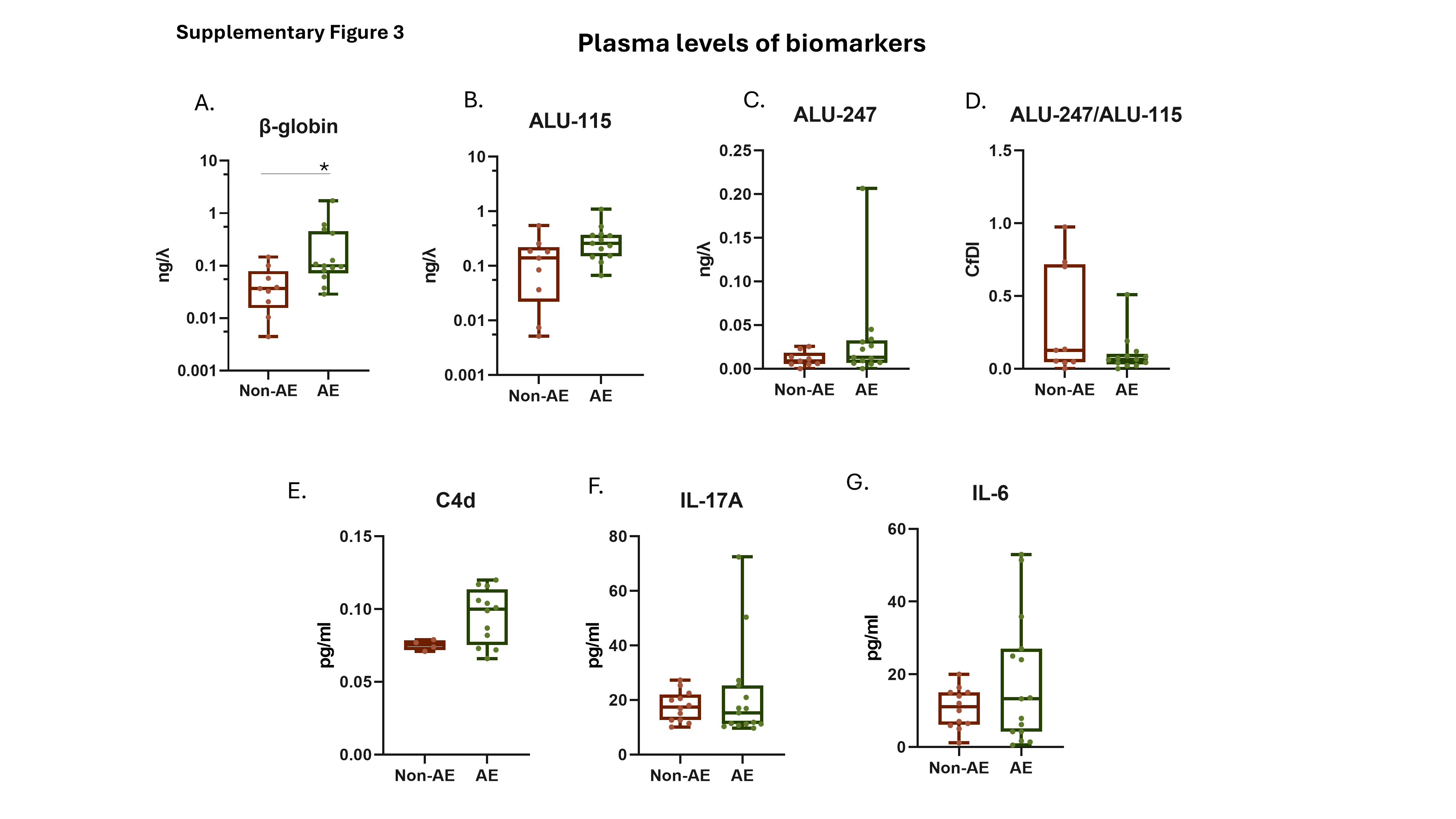

Supplement: Supplementary Figure 3 — Plasma levels of cell-free DNA, DNA integrity, inflammatory cytokines, and C4d between controls and patients with autoimmune encephalitis. Statistically significant results are depicted, *p<0.05. AE, autoimmune encephalitis; non-AE, controls and disease controls. [file Image3.tif]

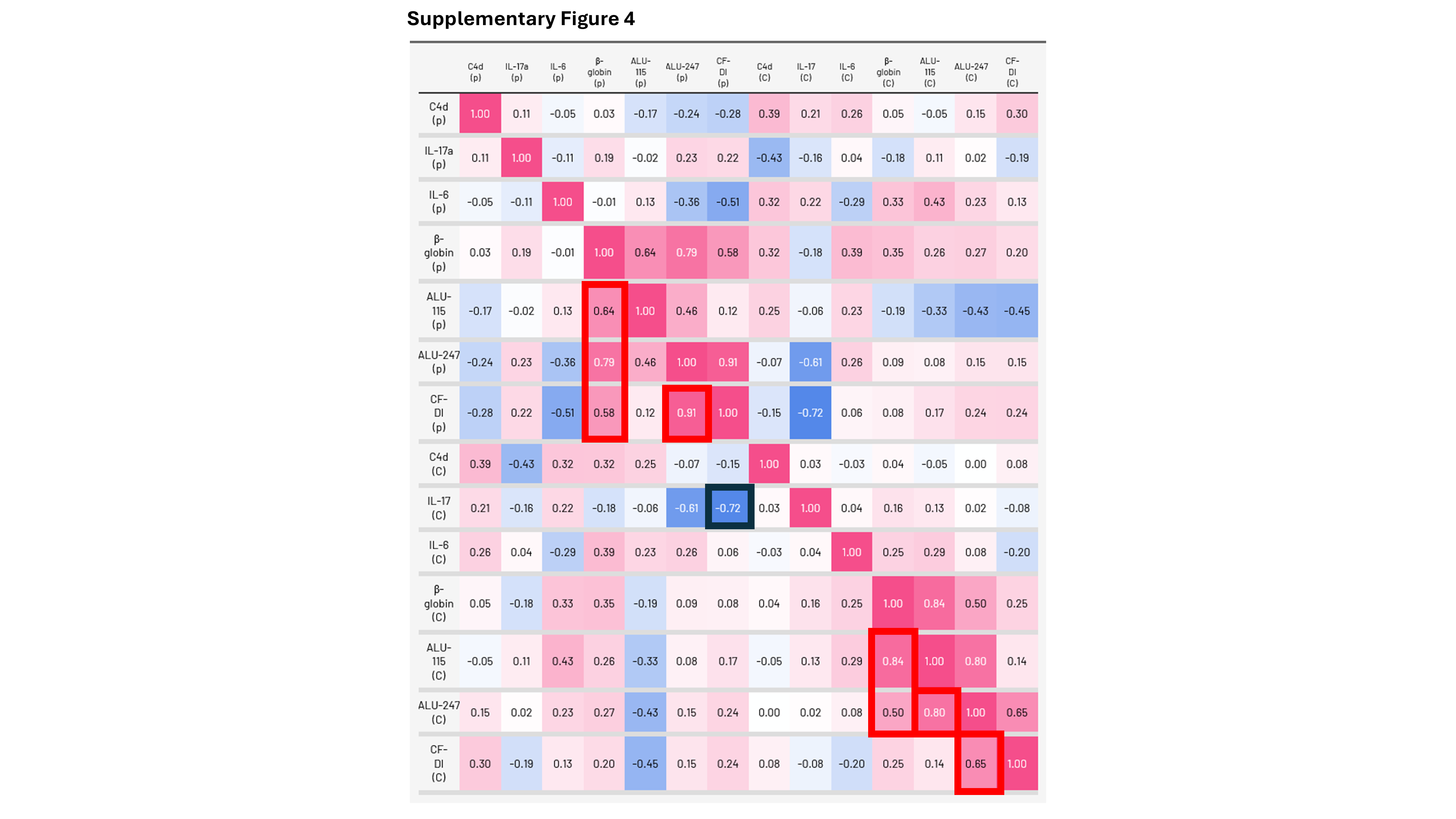

Supplement: Supplementary Figure 4 — Correlation matrix of all parameters in CSF and plasma measured in patients in all types of autoimmune encephalitis. In the correlation matrix the Spearman R is depicted, and color intensity corresponds to the magnitude of the correlation (negative: blue colors, positive: purple). C, cerebrospinal fluid; p, plasma; CFDI, cell free DNA integrity. Image was generated using Flourish (https://flourish.studio). [file Image4.tif]
